# Cytochrome P450-soluble epoxide hydrolase derived linoleic acid oxylipins and cognitive performance in type 2 diabetes

**DOI:** 10.1016/j.jlr.2023.100395

**Published:** 2023-05-26

**Authors:** Natasha Z. Anita, Felicia Kwan, Si Won Ryoo, Chelsi Major-Orfao, William Z. Lin, Shiropa Noor, Krista L. Lanctôt, Nathan Herrmann, Paul I. Oh, Baiju R. Shah, Jeremy Gilbert, Angela Assal, Ilana J. Halperin, Ameer Y. Taha, Walter Swardfager

**Affiliations:** 1 Department of Pharmacology & Toxicology, University of Toronto, Toronto, ON, Canada; 2 Sunnybrook Research Institute, Toronto, ON, Canada; 3 KITE Research Institute, Toronto Rehabilitation Institute-University Health Network, Toronto, ON, Canada; 4 Department of Psychiatry, University of Toronto, Toronto, ON, Canada; 5 Sunnybrook Health Sciences Centre, Toronto, ON, Canada; 6 Department of Food Science and Technology, College of Agriculture and Environmental Sciences, University of California, Davis, CA, USA; 7 West Coast Metabolomics Center, Genome Center, University of California, Davis, Davis, CA, USA; 8 Center for Neuroscience, University of California, Davis, Davis, CA, USA

**Keywords:** Alzheimer’s disease, inflammation, lipids, obesity, oxidized lipids

## Abstract

Type 2 diabetes mellitus (T2DM) increases the risk of cognitive decline and dementia. Disruptions in the cytochrome P450-soluble epoxide hydrolase (CYP450-sEH) pathway have been reported in T2DM, obesity and cognitive impairment. We examine linoleic acid (LA)-derived CYP450-sEH oxylipins and cognition in T2DM and explore potential differences between obese and nonobese individuals. The study included 51 obese and 57 nonobese participants (mean age 63.0 ± 9.9, 49% women) with T2DM. Executive function was assessed using the Stroop Color-Word Interference Test, FAS-Verbal Fluency Test, Digit Symbol Substitution Test, and Trails Making Test-Part B. Verbal memory was assessed using the California Verbal Learning Test, second Edition. Four LA-derived oxylipins were analyzed by ultra-high-pressure–LC/MS, and the 12,13-dihydroxyoctadecamonoenoic acid (12,13-DiHOME) considered the main species of interest. Models controlled for age, sex, BMI, glycosylated hemoglobin A1c, diabetes duration, depression, hypertension, and education. The sEH-derived 12,13-DiHOME was associated with poorer executive function scores (F_1,98_ = 7.513, *P* = 0.007). The CYP450-derived 12(13)-epoxyoctadecamonoenoic acid (12(13)-EpOME) was associated with poorer executive function and verbal memory scores (F_1,98_ = 7.222, *P* = 0.008 and F_1,98_ = 4.621, *P* = 0.034, respectively). There were interactions between obesity and the 12,13-DiHOME/12(13)-EpOME ratio (F_1,97_ = 5.498, *P* = 0.021) and between obesity and 9(10)-epoxyoctadecamonoenoic acid (9(10)-EpOME) concentrations (F_1,97_ = 4.126, *P* = 0.045), predicting executive function such that relationships were stronger in obese individuals. These findings suggest that the CYP450-sEH pathway as a potential therapeutic target for cognitive decline in T2DM. For some markers, relationships may be obesity dependent.

Type 2 diabetes mellitus (T2DM) is associated with a more rapid decline in cognitive performance with aging and with an increased risk of developing cognitive impairment and dementia (1). While the underlying mechanisms remain unclear, cognitive impairment among people with T2DM has been associated with systemic inflammation, oxidative stress, mitochondrial dysfunction, and insulin resistance (2, 3). The inflammatory process in T2DM is multifactorial, involving changes in cytokines and in inflammatory and proresolving lipid mediators known as oxylipins (4, 5). The cytochrome P450-soluble epoxide hydrolase (CYP450-sEH) pathway generates oxylipins from parent polyunsaturated fatty acids, and it has been hypothesized to play a role in both T2DM and cognitive decline (5, 6, 7, 8). The majority of this evidence stems from animal work (reviewed and diagrammed in (8)), emphasizing the need for clinical studies. The pathway has also been associated with obesity (9), which is common but not ubiquitous in this population (10).

Polyunsaturated fatty acids can be metabolized by CYP450s (primarily CYP2J2s and CYP2Cs) into epoxide species, many of which are anti-inflammatory and potentially proresolving (11, 12). However, epoxides may be limited in their activities by sEH, an enzyme that catalyzes their hydrolysis into less active and potentially cytotoxic vicinal diol species. The functions of the diols are unclear; however, linoleic acid (LA)-derived diols (i.e., 12,13-dihydroxyoctadecamonoenoic acid [12,13-DiHOME] and 9,10-dihydroxyoctadecamonoenoic acid [9,10-DiHOME]) have been linked to cytotoxicity (13). Little is known about the physiological role of LA-derived epoxides 9(10)-epoxyoctadecamonoenoic acid (9(10)-EpOME) and 12(13) epoxyoctadecamonoenoic acid (12(13)-EpOME) (14). The diols derived from these species have been suggested recently to have endocrine functions (15); however, the 12,13-DiHOME or its ratio with the 12(13)-EpOME, and the 9,10-DiHOME/9(10)-EpOME ratio were associated with poorer performance on cognitive tests of executive function in groups of older individuals not selected to have T2DM (6, 7, 16).

It has been noted that individuals with T2DM had lower levels of LA-derived epoxides compared to healthy controls (5), while individuals with AD and T2DM have been shown to have higher levels of serum diols compared to cognitively normal people with T2DM (17), but it remains unknown whether these species are related to specific cognitive outcomes in this population.

This study investigates the association between serum LA CYP450/sEH-derived oxylipins and cognitive performance in individuals with T2DM who are cognitively normal from a clinical perspective. We hypothesized that LA diols would be associated with poor executive function as observed previously in people with cerebral small vessel disease (6). We also compare these relationships between obese and nonobese individuals, as obesity is known to affect oxylipin concentrations (18, 19).

## Materials and methods

### Study participants

This cross-sectional study recruited participants from Sunnybrook Health Sciences Centre or the University Health Network Toronto Rehabilitation Institute Cardiac Rehab Program into the Sunnybrook Type 2 Diabetes Study in Ontario, Canada (NCT04455867). All participants were over the age of 18 and diagnosed with either prediabetes (glycated hemoglobin [HbA1c] between 6.0% and 6.4%) or T2DM (HbA1c above 6.4%, impaired fasting glucose or impaired glucose tolerance). Exclusion criteria included type 1 diabetes, neurological or neurodegenerative diagnoses (i.e., mild cognitive impairment, Alzheimer’s disease, dementia, stroke, etc.), pregnancy, active cancer, prior diagnoses of schizophrenia or bipolar disorder, current or past substance use disorder (within the last 5 years, excluding nicotine use), poor score on the Mini Mental Status Exam (total score <24) (20) and/or inability to provide informed consent. Ethics approval was given by the Research Ethics Board in accordance with the Declaration of Helsinki, and all study participants provided written informed consent prior to their inclusion.

### Demographics, clinical characteristics, and measurements

Standardized interviews were conducted to collect demographic, clinical, and medical data during study assessments. Medical charts were also reviewed in the event of missing data. Body mass index (BMI) was calculated using weight and height measurements obtained by study or clinical staff. Obesity was defined as BMI ≥30 kg/m^2^. Fasting blood glucose was measured via glucometer (Bayer, Mississauga, ON, Canada). HbA1c, triglycerides, and cholesterol profiles were obtained with standard clinical measures from fasting blood samples.

### Serum measurements

Blood samples were drawn following a 9-h overnight fast and centrifuged at 1000 *g* for 10 min at 23°C. Serum was separated and stored at −80°C until the time of the analysis. A total of four LA-derived oxylipin species were analyzed by ultra-high-pressure liquid chromatography tandem mass spectrometry as described previously (6, 21): 9(10)-EpOME, 12(13)-EpOME, 9,10-DiHOME, and 12,13-DiHOME. The 12,13-DiHOME was considered the main species of interest and the others exploratory. An internal standard per oxylipin was used for the quantitative evaluation. The limit of detection was estimated using the lowest observable point on the standard calibration curve with a signal to noise ratio of three or more.

### Cognitive assessments

Cognitive assessments were administered by trained researchers using a standardized battery of tests based on Canadian Stroke Network & National Institutes of Neurological Disorders recommended 30-min battery (22). The battery has been used extensively in people with T2DM and found to be sensitive to determinants of cognitive health in this population (23, 24). Executive function was assessed using the Victoria Version of the Stroop Color-Word Interference Test (Stroop Test-Color), FAS verbal fluency test, Digit Symbol Substitution Test, and Trails Making Test Part B. Each of these tests were evaluated using age-matched standard scores and unit weighted z-scores, which were combined then standardized to provide a composite executive function score. Verbal memory was assessed using the California Verbal Learning Test second Edition. A composite verbal memory score was calculated using the z-score measures from Learning Trials 1–5 (Verbal Learning), short-delay free recall (short-term memory), and long-delay free recall (long-term memory). Each of these tests were evaluated using age, sex, and education-matched unit weighted z-scores, which were combined then standardized to provide a composite verbal memory score (22).

### Depressive episode diagnosis

Current depressive episodes (i.e., at the time of study participation) were identified using the Structured Clinical Interview for DSM-5 criteria, Research Version (25, 26). To be classified as depressed, individuals needed to have at least one of diminished mood or decreased interest/pleasure and at least four other depressive symptoms lasting nearly every day for a period of at least 2 weeks.

Depression was measured as people with T2DM are at an increased risk of developing depressive symptoms (27), which are associated with cognitive deficits (28). Perturbations in the CYP450-sEH pathway have been implicated in rodent models of depression (29), and differences in serum diol and epoxide concentrations have also been reported between depressed and nondepressed states in individuals with T2DM (21) and seasonal depression (30).

### Statistical analysis

All analyses were conducted using IBM SPSS, version 28. If skewness or kurtosis was detected, oxylipins were log-transformed prior to analyses. Analyses of covariance were used to determine the association of LA-derived oxylipins with executive function and verbal memory composite scores, adjusting for covariates that may affect the association. Missing oxylipin values were imputed using the “imputeLCMD” package in R (31). Potential confounders to the relationship between cognition and oxylipins, included age, sex, BMI, HbA1c, diabetes duration, depression status, hypertension, and years of education, which were included as covariates a priori. These models were bootstrapped with 1000 iterations of random sampling to increase confidence in any statistical findings. To test potential interaction effects between LA-derived oxylipins and obesity on cognitive outcomes, analyses of covariance models controlling the same covariates except BMI were used, wherein obesity status (either obese or nonobese based on BMI ≥ 30 kg/m^2^) and an obesity × oxylipin interaction were added as independents. Scatterplots were generated using the “ggplot2” package (32).

## Results

### Participant characteristics and measurements

**Fig. 1:**
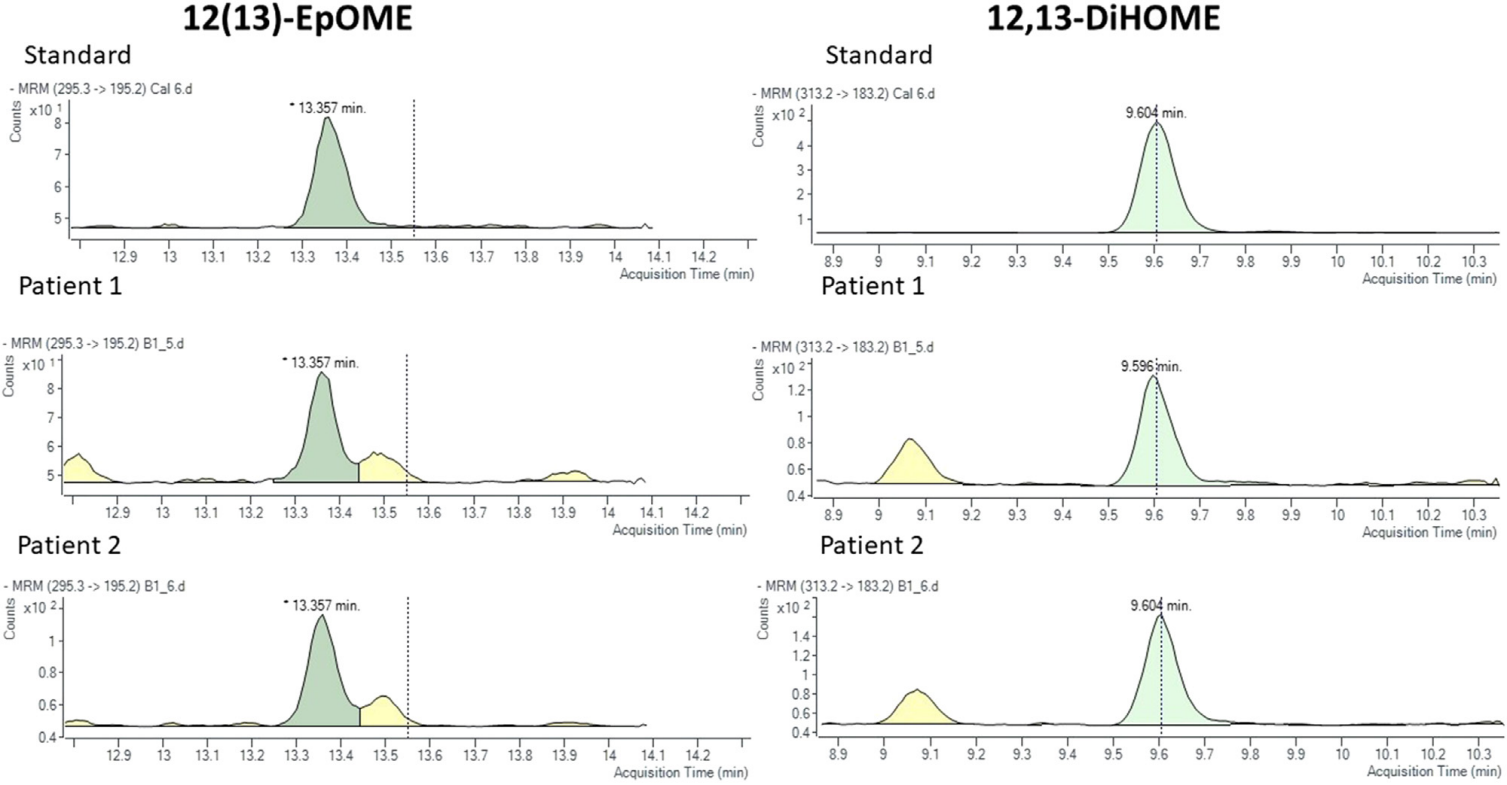
Representative chromatograms for 12(13)-epoxyoctadecamonoenoic acid (12(13)-EpOME; left) and 12,13-dihydroxyoctadecamonoenoic acid (12,13-DiHOME; right) depicting the standard and two sample patients for the ultra-high pressure–liquid chromatography tandem mass spectrometry.

**Fig. 2:**
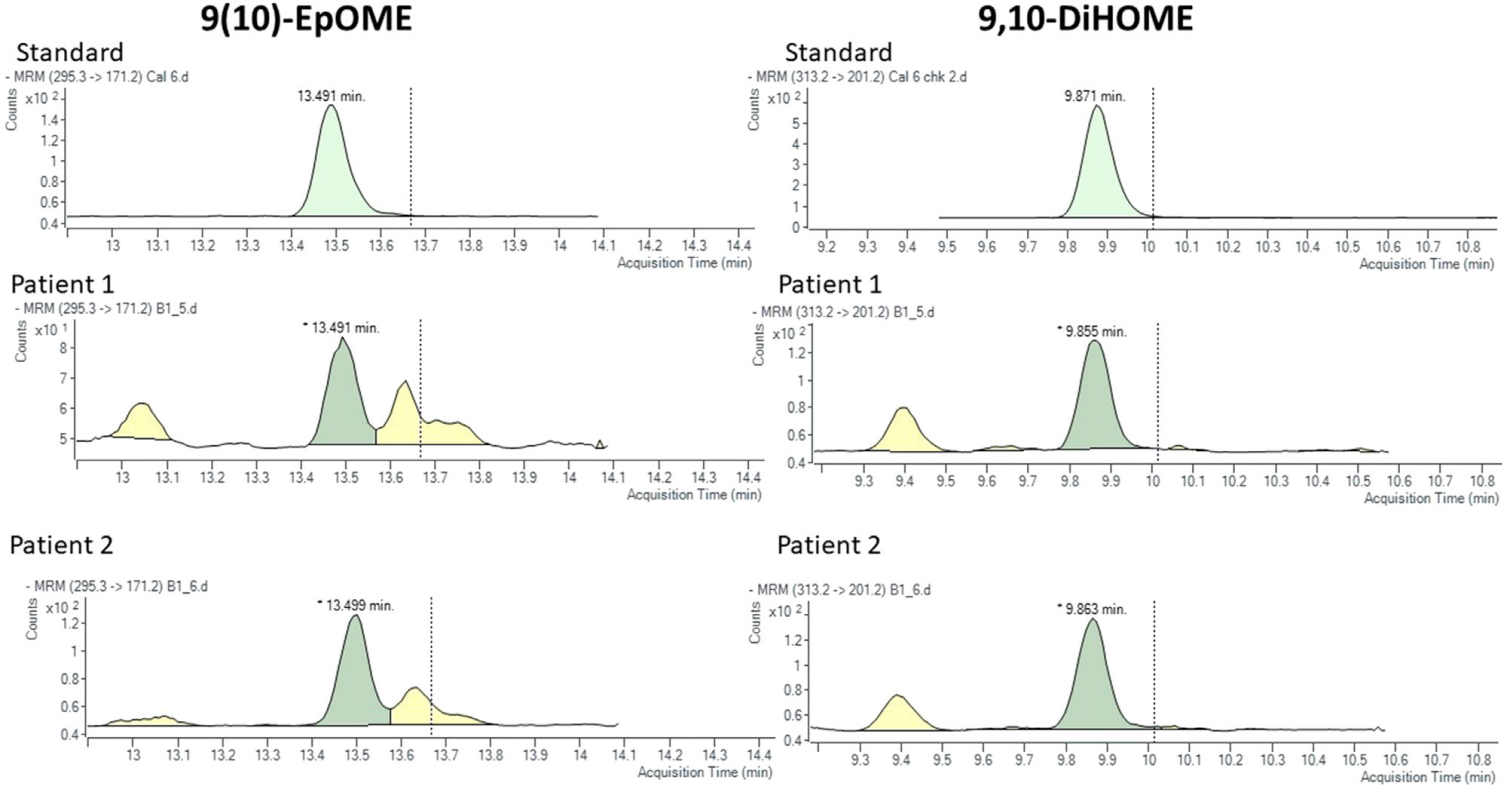
Representative chromatograms for 9(10)-epoxyoctadecamonoenoic acid (9(10)-EpOME; left) and 9,10-dihydroxyoctadecamonoenoic acid (9,10-DiHOME; right) depicting the standard and two sample patients for the ultra-high pressure–liquid chromatography tandem mass spectrometry.

After applying exclusion criteria, 108 individuals were included in the analysis. Participant characteristics are summarized in Table 1. Individuals with obesity (BMI ≥ 30 kg/m^2^) were identified and compared to nonobese individuals. The medians and interquartile ranges for serum oxylipins are summarized in Table 2. The limit of detection ranged from 0.12–1.95 nM across the four species (see Fig. 1, Fig. 2). The means and standard deviations for each cognitive test are summarized in Table 3.

**Table 1:** Participant characteristics for the T2DM group and obese and nonobese subgroups

Characteristic	T2DM (n = 108)	Obese group (n = 51)	Nonobese group (n = 57)	*P*
Demographics				
Age (years)	63.0 ± 9.9	61.7 ± 10.4	64.2 ± 9.3	0.179
Sex (%female)	53 (49.1%)	26 (51.0%)	27 (47.4%)	0.708
BMI (kg/m^2^)	31.1 ± 6.5	36.3 ± 5.5	26.4 ± 2.8	**<0.001**
Weight (kg)	88.8 ± 23.5	103.6 ± 18.9	75.2 ± 18.7	**<0.001**
Diabetes duration (years)	7.8 ± 9.9	6.4 ± 10.0	9.1 ± 9.8	0.175
Education (years)	16.0 ± 2.9	15.7 ± 3.3	16.3 ± 2.5	0.259
Smoking (% current)	4 (3.7%)	3 (5.9%)	1 (1.8%)	0.257
Working status (%employed)	47 (43.5%)	20 (39.2%)	27 (47.4%)	0.394
Clinical measures				
HbA1c (%)	7.4 ± 1.2	7.5 ± 1.3	7.2 ± 1.1	0.155
Triglycerides (mmol/L)^a^	1.6 ± 1.1	1.6 ± 0.8	1.7 ± 1.3	0.755
HDL (mmol/L)^a^	1.2 ± 0.3	1.2 ± 0.3	1.3 ± 0.4	0.084
LDL(mmol/L)^a^	2.2 ± 0.9	2.2 ± 1.0	2.1 ± 0.8	0.771
Total cholesterol (mmol/L)^a^	4.1 ± 0.9	4.1 ± 1.1	4.1 ± 0.8	0.863
Drinking status^b^				0.348
2+ drinks at least 3 times a week	2 (1.9%)	0 (0.0%)	2 (3.5%)	
3–7 drinks/week	12 (11.1%)	6 (11.8%)	6 (10.5%)	
1–3 drinks/week	27 (25.0%)	11 (21.6%)	16 (28.1%)	
A few per year	46 (42.6%)	26 (51.0%)	20 (35.1%)	
Never	21 (19.4%)	8 (15.7%)	13 (22.8%)	
Ethnicity^c^				0.055
Caucasian	75 (69.4%)	40 (78.4%)	35 (61.4%)	
Middle Eastern	3 (2.8%)	0 (0.0%)	3 (5.3%)	
Asian	10 (9.3%)	2 (3.9%)	8 (14.0%)	
South Asian	11 (10.2%)	2 (3.9%)	9 (15.8%)	
Black	8 (7.4%)	6 (11.8%)	2 (3.5%)	
Indigenous	1 (0.9%)	1 (2.0%)	0 (0.0%)	
Diabetes complications				
Nephropathy	9 (8.3%)	4 (7.8%)	5 (8.8%)	0.862
Retinopathy	8 (7.4%)	3 (5.9%)	5 (8.8%)	0.567
Neuropathy	24 (22.2%)	13 (25.5%)	11 (19.3%)	0.440
Comorbidities				
Sleep apnea	13 (12.0%)	10 (19.6%)	3 (5.3%)	**0.022**
Renal disease	5 (4.6%)	2 (3.9%)	3 (5.3%)	0.740
Hypertension	63 (58.3%)	38 (74.5%)	25 (43.9%)	**0.001**
Coronary artery disease	5 (4.6%)	3 (5.9%)	2 (3.5%)	0.558
Myocardial infarction	7 (6.5%)	3 (5.9%)	4 (7.0%)	0.811
CABG	8 (7.4%)	2 (3.9%)	6 (10.5%)	0.191
Dyslipidemia	67 (62.0%)	34 (66.7%)	33 (57.9%)	0.348
Psychiatric conditions				
Current depression	17 (15.7%)	11 (21.6%)	6 (10.5%)	0.116
Anxiety	11 (10.2%)	6 (11.8%)	5 (8.8%)	0.608
Medications				
Antidepressant	17 (15.7%)	9 (17.6%)	8 (14.0%)	0.607
Anxiolytic	1 (0.9%)	1 (2.0%)	0 (0.0%)	0.288
Aspirin	28 (25.9%)	14 (27.5%)	14 (24.6%)	0.732
Statins	71 (65.7%)	35 (68.6%)	36 (63.2%)	0.550
Anti-hypertensives	66 (61.1%)	37 (72.5%)	29 (50.9%)	**0.021**
ACE inhibitors	30 (27.8%)	19 (37.3%)	11 (19.3%)	**0.038**
Diuretic	22 (20.4%)	12 (23.5%)	10 (17.5%)	0.441
Angiotensin receptor blockers	21 (19.4%)	11 (21.6%)	10 (17.5%)	0.598
Beta-blockers	25 (23.1%)	15 (29.4%)	10 (17.5%)	0.144
Calcium channel blockers	24 (22.2%)	14 (27.5%)	10 (17.5%)	0.216
Glucocorticoid	3 (2.8%)	1 (2.0%)	2 (3.5%)	0.625
Osteoporosis meds	3 (2.8%)	2 (3.9%)	1 (1.8%)	0.494
Anti-diabetics				
Insulin	22 (20.4%)	11 (21.6%)	11 (19.3%)	0.770
SGLT2 inhibitors	27 (25.0%)	12 (23.5%)	15 (26.3%)	0.738
Incretins	8 (7.4%)	4 (7.8%)	4 (7.0%)	0.870
DPP4 inhibitors	30 (27.8%)	13 (25.5%)	17 (29.8%)	0.616
Sulfonylurea	15 (13.9%)	7 (13.7%)	8 (14.0%)	0.963
Metformin	70 (64.8%)	29 (56.9%)	41 (71.9%)	0.102

Variables are summarized as mean ± standard deviation or percentage. Entries in bold are significantly different at an uncorrected *P*-value < 0.05 between the obese and the non-obese group. Continuous variables were compared with *t*-tests, and categorical variables were compared using chi^2^ tests.

BMI, body mass index; DPP-4 inhibitor, dipeptidyl peptidase-4 inhibitor; HbA1c, glycated hemoglobin; SGLT2 inhibitor, sodium-glucose transport protein 2 inhibitor; T2DM, type 2 diabetes mellitus.

a Lipid measures were not known for four participants (three in obese group, one in nonobese group). These are the means for available data (n = 104).

b Chi^2^ test performed with 1 degree of freedom (frequent alcohol use vs. nonuse).

c Chi^2^ test performed with 1 degree of freedom (Caucasian vs. other).

**Table 2:** Serum oxylipin measures of study participants

Oxylipin	Total detectability	T2DM (n = 108)^a^	Obese group (n = 51)^a^	Non-obese group (n = 57)^a^	*P*
9(10)EpOME	97%	3.5 (3.4)	3.4 (3.4)	3.7 (3.9)	0.137
12(13)EpOME	100%	9.7 (10.5)	9.7 (11.1)	9.8 (10.5)	0.412
9,10-DiHOME	94%	1.9 (1.8)	1.9 (1.7)	2.1 (2.2)	0.898
12,13-DiHOME	97%	3.6 (2.7)	3.8 (2.6)	3.3 (2.8)	0.839

Oxylipins were log-transformed prior to *t*-tests.

12,13-DiHOME, 12,13-dihydroxyoctadecamonoenoic acid; 12(13)EpOME, 12(13)-Epoxyoctadecamonoenoic acid; 9,10-DiHOME, 9,10-Dihydroxyoctadecamonoenoic acid; 9(10)EpOME, 9(10)-Epoxyoctadecamonoenoic acid; T2DM, type 2 diabetes mellitus.

a Expressed as median (interquartile range), nM.

**Table 3:** Cognitive outcomes of study participants

Cognitive measures	T2DM (n = 108)	Obese group (n = 51)	Nonobese group (n = 57)	*t*	*P*
Executive Function					
Stroop Color-Word Interference Test^a^					
Seconds	32.0 ± 11.1	33.6 ± 11.8	30.5 ± 10.3		
Z-score	−0.2 ± 1.0	−0.4 ± 0.9	0.0 ± 1.0	−2.005	**0.046**
FAS Verbal Fluency Test					
Words	34.3 ± 11.4	33.4 ± 11.3	35.0 ± 11.5		
Z-score	−0.3 ± 1.0	−0.4 ± 1.0	−0.2 ± 1.0	−1.034	0.313
Digit Symbol Substitution Test					
Symbols	56.0 ± 13.7	54.8 ± 15.1	57.1 ± 12.3		
Z-score	−0.2 ± 0.8	−0.4 ± 0.8	−0.1 ± 0.8	−1.782	0.077
Trails Making Test Part B					
Seconds	103.4 ± 54.8	105.1 ± 48.9	101.9 ± 60.0		
Z-score	−0.4 ± 0.9	−0.5 ± 1.0	−0.4 ± 0.9	−0.717	0.475
Composite					
Z-score	0.0 ± 1.0	−0.2 ± 0.9	0.2 ± 1.0	−1.950	0.058
Verbal memory					
CVLT-II Learning Trials 1–5					
Words	44.2 ± 11.7	43.0 ± 11.4	45.3 ± 11.9		
Z-score	0.1 ± 1.1	−0.1 ± 1.1	0.2 ± 1.2	−1.620	0.094
CVLT-II Short Delay Free Recall					
Words	9.0 ± 3.5	8.6 ± 3.3	9.4 ± 3.5		
Z-score	0.0 ± 1.1	−0.2 ± 1.0	0.2 ± 1.1	−1.963	**0.049**
CVLT-II Long Delay Free Recall					
Words	9.6 ± 3.8	9.3 ± 3.4	9.9 ± 4.1		
Z-score	0.0 ± 1.2	−0.2 ± 1.1	0.1 ± 1.2	−1.325	0.192
Composite					
Z-score	0.0 ± 1.0	−0.2 ± 0.9	0.2 ± 1.0	−1.761	0.089

Composite z-scores were used for comparing obese and nonobese groups. Entries in bold are significantly different at an uncorrected *P*-value < 0.05 between the obese and the non-obese group.

CVLT-II, California Verbal Learning Test second edition; T2DM, Type 2 diabetes mellitus.

a Stroop measure was missing in two individuals (1 obese, 1 non-obese).

### Associations between oxylipins and executive function

**Fig. 3:**
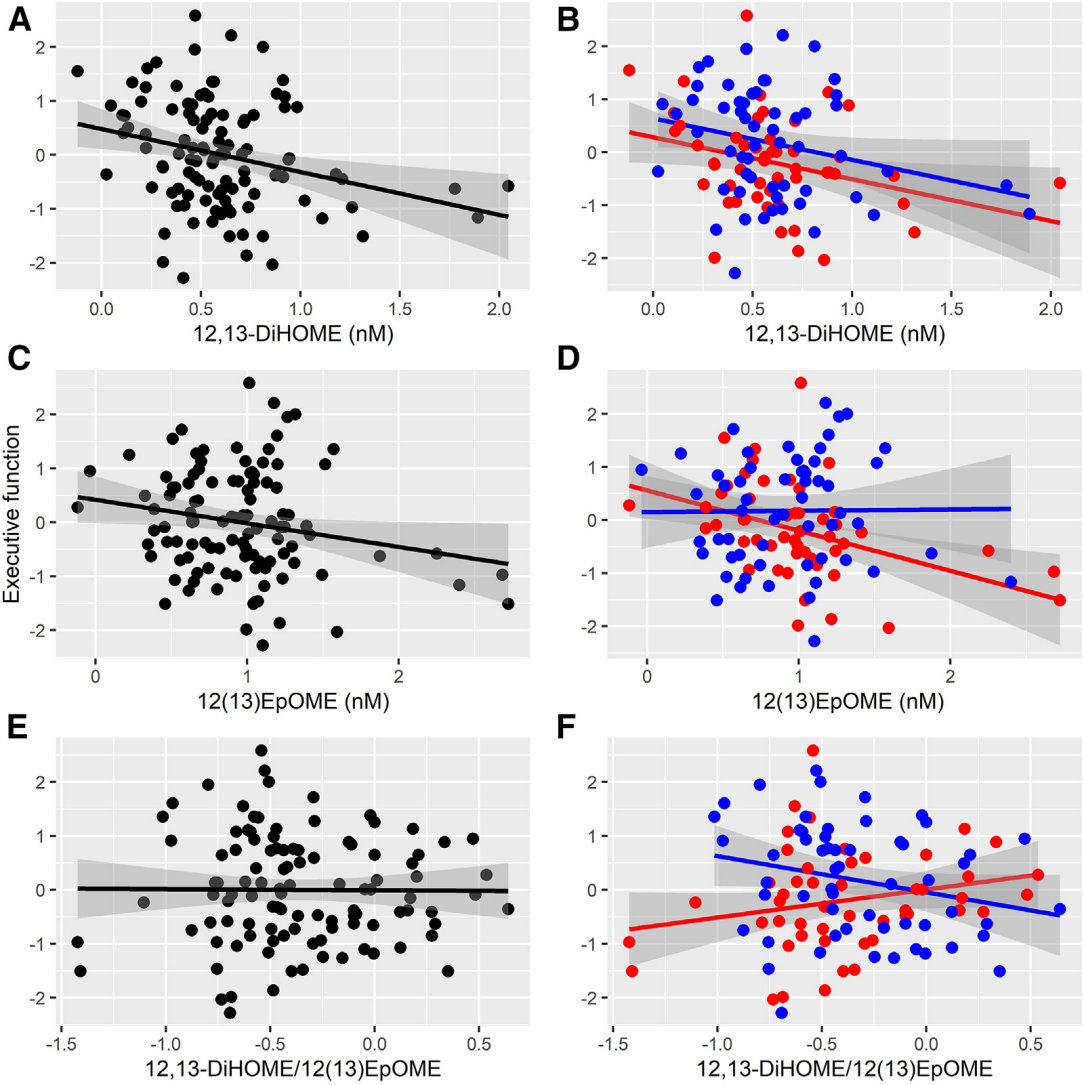
Associations between the 12,13-dihydroxyoctadecamonoenoic acid (12,13-DiHOME) species and executive function composite score in the (A) entire group and (B) broken down by obese (red) and nonobese (blue) subgroups; associations between the 12(13) epoxyoctadecamonoenoic acid (12(13)-EpOME) species and executive function composite score in the (C) entire group and (D) broken down by obese (red) and nonobese (blue) subgroups; associations between the 12,13-DiHOME/12(13)EpOME ratio and executive function composite score in the (E) entire group and (F) broken down by obese (red) and nonobese (blue) subgroups.

**Fig. 4:**
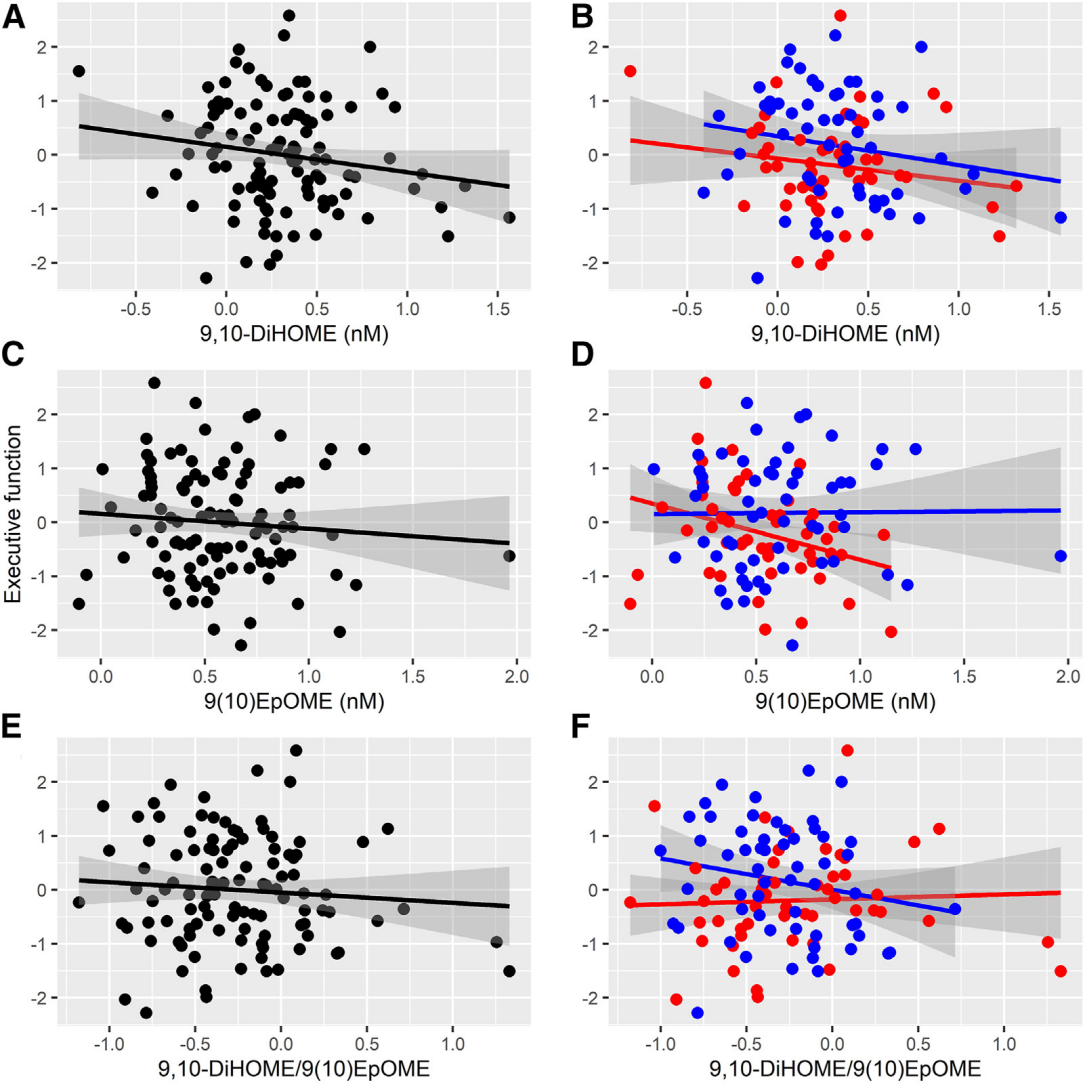
Associations between the 9,10-dihydroxyoctadecamonoenoic acid (9,10-DiHOME) species and executive function composite score in the (A) entire group and (B) broken down by obese (red) and nonobese (blue) subgroups; associations between the 9(10)-epoxyoctadecamonoenoic acid (9(10)-EpOME) species and executive function composite score in the (C) entire group and (D) broken down by obese (red) and nonobese (blue) subgroups; associations between the 9,10-DiHOME/9(10)EpOME ratio and executive function composite score in the (E) entire group and (F) broken down by obese (red) and nonobese (blue) subgroups.

In the adjusted model, 12,13-DiHOME was associated with poorer executive function composite scores (F_1,98_ = 7.513, *P* = 0.007; see Fig. 3A). 12(13)-EpOME was also associated with lower executive function composite scores (F_1,98_ = 7.222, *P* = 0.008; see Fig. 3C). No associations were observed for 9,10-DiHOME (F_1,98_ = 3.016, *P* = 0.086; see Fig. 4A) or 9(10)-EpOME (F_1,98_ = 2.368, *P* = 0.127; see Fig. 4C) in the adjusted models. No associations were observed for the diol/epoxide ratios 12,13-DiHOME/12(13)-EpOME (F_1,98_ = 0.463, *P* = 0.498; see Fig. 3E) or 9,10-DiHOME/9(10)-EpOME (F_1,98_ = 0.138, *P* = 0.711; see Fig. 4E).

### Associations between oxylipins and verbal memory

In an adjusted model, 12(13)-EpOME was associated with lower verbal memory composite scores (F_1,98_ = 4.621, *P* = 0.034; see supplemental Fig. S1C). No associations were observed for 12,13-DiHOME (F_1,98_ = 1.900, *P* = 0.171), 9,10-DiHOME (F_1,98_ = 1.713, *P* = 0.194), or 9(10)-EpOME (F_1,98_ = 0.032, *P* = 0.859) (see supplemental Fig. S1A, B, and D).

No associations were observed for the diol/epoxide ratios 12,13-DiHOME/12(13)-EpOME (F_1,98_ = 1.732, *P* = 0.191) or 9,10-DiHOME/9(10)-EpOME (F_1,98_ = 0.985, *P* = 0.324) (see supplemental Fig. S1E).

### Interactions between oxylipins and obesity on executive function

No interaction was observed between obesity and 12,13-DiHOME concentrations (F_1,97_ = 0.002, *P* = 0.969; see Fig. 3B). There was a trend toward and interaction between 12(13)-EpOME and obesity in predicting lower executive function scores (F_1,97_ = 3.411, *P* = 0.068; see Fig. 3D), such that the association trended more strongly in the obese group. There was a significant interaction between the 12,13-DiHOME/12(13)-EpOME ratio and obesity such that the ratio was associated with higher executive function scores in the obese group (F_1,97_ = 5.498, *P* = 0.021; see Fig. 3F).

There was a significant interaction between 9(10)-EpOME concentrations and obesity such that the 9(10)-EpOME was associated with lower executive function scores in obese individuals (F_1,97_ = 4.126, *P* = 0.045; see Fig. 4D). No interactions were observed for the 9,10-DiHOME (F_1,97_ = 0.067, *P* = 0.797; see Fig. 4B) or the 9,10-DiHOME/9(10)-EpOME ratio (F_1,97_ = 1.792, *P* = 0.184; see Fig. 4F).

### Interactions between oxylipins and obesity on verbal memory

No interaction was observed between obesity and 12(13)-EpOME (F_1,97_ = 0.004, *P* = 0.950; see supplemental Fig. S1D), 12,13-DiHOME (F_1,97_ = 0.150, *P* = 0.699), or the 12,13-DiHOME/12(13)-EpOME ratio (F_1,97_ = 0.271, *P* = 0.604) in the adjusted models (see supplemental Fig. S1B, F).

No interactions with obesity were observed for the 9,10-DiHOME (F_1,97_ = 0.173, *P* = 0.678), 9(10)-EpOME (F_1,97_ = 0.657, *P* = 0.420), or the 9,10-DiHOME/9(10)-EpOME ratio (F_1,97_ = 0.004, *P* = 0.950; see supplemental Fig. S2B, D, and F).

### Posthoc models and sensitivity analyses

Because more obese participants had sleep apnea and antihypertensive use, executive function analyses were repeated in posthoc models controlling for these additional covariates. All executive function results were similar in these posthoc models (see supplemental Results 1.1 for sleep apnea, supplemental Results 1.2 for antihypertensive use).

The 12,13-DiHOME, 9,10-DiHOME, and 9(10)-EpOME species had some missing cases (see Table 2 for detectability), thus executive function analyses were repeated after excluding these missing samples for each species. These results were generally consistent with the main analyses. In addition to the 12(13)-EpOME, the 9(10)-EpOME was also associated with lower executive function in the sensitivity analyses (see supplemental Results 1.3).

## Discussion

This study explored the relationships between LA-derived oxylipin species and cognitive outcomes in individuals with T2DM. The sEH-derived 12,13-DiHOME was associated with lower executive function composite scores. The CYP450-derived 12(13)-EpOME was also associated with lower executive function composite scores, and this relationship was stronger in obese individuals. Interestingly, the 12,13-DiHOME/12(13)-EpOME ratio was associated with higher executive function in the obese group. The present results suggest the potential role of LA CYP450-sEH-derived species in cognitive performance in people with T2DM, which may be influenced by obesity.

The relationship between this sEH-derived diol is broadly consistent with previous studies in people without T2DM. In people with hypertension, the plasma ratio of LA-derived 9,10-DiHOME/9(10)-EpOME was associated with lower executive function scores (7), while the serum 12,13-DiHOME/12(13)-EpOME ratio was associated with lower perceptual speed in cognitively normal individuals and lower executive function in people with a history of transient ischemic attack (6, 16). In the latter study, higher 12,13-DiHOME concentrations were found in people who had extensive cerebral small vessel disease, as indicated by elevated white matter hyperintensities (WMHs) (6). The 12,13-DiHOME/12(13)-EpOME ratio was also associated with larger WMH volumes in a stroke population (33). Although not measured here, WMHs have been associated with impairments in processing speed, attention, and executive function in T2DM (34). Higher sEH-derived diols have also been reported in postmortem brain samples from individuals with vascular cognitive impairment (35). The present work adds to those studies, suggesting the relevance of sEH-derived diols in cognition among individuals with T2DM. sEH inhibitors have already been shown to improve cognitive symptoms in rodent models of T2DM (36, 37), and clinical trials are needed to determine whether similar results may be observed in humans.

In this study, the CYP450-derived epoxide 12(13)-EpOME was associated with poorer executive performance. This result contrasts with previous reports where epoxides of LA were not associated with executive function (6, 7). The reason for this discrepancy is unclear; however, previous studies did not select populations to have diabetes or obesity. The 12(13)-EpOME was also associated with poorer verbal memory scores. These findings differ from a previous study in people with transient ischemic attack where no association with verbal memory was seen (6). These associations were smaller than those reported for executive function, though they suggest that the CYP450-sEH pathway may be relevant to verbal memory in T2DM. It is possible that these markers could reflect increased intake of LA, inflammation, or other processes, which might be relevant to hippocampal function or neurodegeneration in addition to small vessel disease, warranting further study in T2DM.

In the current study, both LA epoxides 12(13)-EpOME and 9(10)-EpOME were more strongly associated with poorer executive performance in obese individuals compared to nonobese individuals. Obesity is common in people with T2DM, and it has been linked to metabolic dysfunction, insulin resistance, and dyslipidemia (10). It has been demonstrated that obese people with T2DM had lower cortical thickness and poorer verbal memory scores compared to their nonobese counterparts (38), which raises the possibility that obesity may alter the relationship between T2DM and cognitive deterioration.

Previously, Khadir *et al.* (9) reported higher mRNA and protein levels of *Ephx2* (the gene which encodes the sEH protein) in subcutaneous adipose tissue and peripheral blood mononuclear cells obtained from obese people without T2DM compared to samples from normal-weight controls. It is possible that obesity may alter the sEH pathway in target tissues and consequently modify the relationship between circulating oxylipin species and cognitive outcomes. Endoplasmic reticulum (ER) stress markers such as glucose regulated protein 78 and activating transcription factor 6 were also elevated among obese individuals in that study, indicating potential underlying mechanisms. The ER stress pathway has previously been linked to diabetic neuropathy in rodents, where administering an sEH inhibitor alleviated neuropathic pain and decreased ATF4 and activating transcription factor 6 mRNA levels (39), suggesting potential crosstalk between ER stress and sEH pathways in diabetic complications. Further studies might examine these pathways in cerebral small vessels in T2DM and obesity.

In the present study, the 12,13-DiHOME/12(13)-EpOME ratio, an in vivo marker reflecting sEH metabolic flux, was not associated with cognitive outcomes in T2DM; however, this ratio was associated with higher executive performance in the obese subgroup. This association was driven by the epoxide being associated with lower executive function in the obese subgroup. These results contrast with previous studies that found the 12,13-DiHOME/12(13)-EpOME and 9,10-DiHOME/9(10)-EpOME ratios were associated with poorer executive performance in people with cerebral small vessel disease (6) or hypertension (7). Those studies did not select for people with T2DM, and they did not examine the effect of BMI. Other studies reported higher epoxide concentrations in obese or T2DM populations compared to healthy controls (5, 9). The current study did not find significant differences in serum LA epoxide concentrations between obese versus nonobese individuals with T2DM, although the 9(10)-EpOME was numerically higher. Future studies should compare obese and nonobese individuals with and without T2DM for potential differences in peripheral oxylipin concentrations. The current findings suggest caution when interpreting peripheral blood diol/epoxide ratios in people with obesity and/or T2DM.

It is significant to note that both animal and human work have linked the CYP450-sEH pathway to depression (21, 29, 30). In the present study, the proportion of individuals with current depression did not significantly differ between obese and nonobese individuals with T2DM nor was depression found to be a significant confounder. Nonetheless, given that obesity itself is associated with increased likelihood of depression (40) and cognitive decline (38), future work examining the potential interplay between obesity, T2DM, and the CYP450-sEH pathway would be of great interest.

The current study focused on individuals with T2DM, so the results cannot be generalized to individuals without T2DM. This exploratory study is of a cross-sectional, correlative nature and therefore causation cannot be inferred. Obesity was estimated using BMI, which may not completely account for adiposity. Future studies should investigate alternative measurements of obesity, such as waist-to-hip ratio. The LA-derived species can be elevated in blood due to increased dietary intake of LA, and they have been shown to adversely affect the brain in animal models (41, 42). Future studies should consider potential differences in dietary LA intake and/or peripheral blood LA measurement. The current paper focused only on LA species as these are the most readily detectable in peripheral blood; however, the CYP450-sEH pathway can also act on fatty acids other than LA, such as arachidonic acid, docosahexaenoic acid, and eicosapentaenoic acid, to produce other epoxides and diols which have been linked to cognitive outcomes and WMH (7). Studies accounting for other oxylipin species are warranted.

The current study examined the unesterified oxylipin species as these represent the bioactive form; however, these concentrations may not reflect concentrations of esterified oxylipins (43, 44), which could show additional associations with cognitive outcomes. The oxylipins were also measured from fasted serum samples, and results may differ with nonfasted samples. Borkowski *et al.* (16) previously reported negative associations between serum LA ratios and perceptual speed scores in fasted samples, but not in the nonfasted samples. Additional studies measuring esterified and unesterified species, in fasted and nonfasted states, are needed to better elucidate potential oxylipin differences.

In conclusion, the association between the LA-derived oxylipins and executive function suggests the involvement of CYP450/sEH LA metabolites in cognitive performance among individuals with T2DM and that this association may be modulated by obesity. The diol species may serve as better cognitive biomarkers because differential associations were not found between BMI groups, while the diol to epoxide ratios may need to be interpreted with caution due to potential effects of obesity on the relationship between circulating epoxide concentrations and executive function. Further studies will be required to better understand the relationship between oxylipins with cognitive decline and other complications in people with T2DM and whether the CYP450-sEH pathway may serve as a potential therapeutic target to prevent worsening of cognitive symptoms and subsequent conversion to dementia.

## Data availability

The data pertaining to the current study are available upon request.

## Supplemental data

This article contains supplemental data.

## Conflict of interest

The authors declare the following financial interests/personal relationships which may be considered as potential competing interests. B. R. S. of Sunnybrook Health Sciences Centre is funded by the University of Toronto as the Novo Nordisk Research Chair in Equitable Care of Diabetes and Related Conditions. J. G. of Sunnybrook Health Sciences Centre received speaker honoraria from Abbott, Ascencia, Amgen, Astra Zeneca, Boehringer, Dexcom, Eli Lilly, HLS, Insulet, Janssen, Merck, Novo Nordisk, and Sanofi. J. G. also performs advisory services to Abbott, Amgen, Astra Zeneca, Boehringer, Dexcom, Eli Lilly, Janssen, Merck, Novo Nordisk, and Sanofi. A. A. of Sunnybrook Health Sciences Centre received speaker honoraria from Solutions Event Management. I. J. H. of Sunnybrook Health Sciences Centre received speaker honoraria from Dexcom, Abbott, Sanofi, and Boehringer Ingelheim. These roles are unrelated to the present work. The other authors declare no conflicts of interest.
