# Supplementary material for: Cytochrome P450-soluble epoxide hydrolase derived linoleic acid oxylipins and cognitive performance in type 2 diabetes
Source: J Lipid Res. 2023 May 26;64(7):100395. doi: 10.1016/j.jlr.2023.100395 (PMC10394387; doi:10.1016/j.jlr.2023.100395)
Supplement: Supplemental information [file mmc1.docx]

**SUPPLEMENTAL INFORMATION:**

**Cytochrome p450-soluble epoxide hydrolase derived linoleic acid oxylipins and cognitive performance in type 2 diabetes mellitus**

Natasha Z. Anita^a,b,c^, Felicia Kwan^a,b,c^, Si Won Ryoo ^a,b,c^, Chelsi Major-Orfao ^b,c^, William Z. Lin ^a,b,c^, Shiropa Noor ^a,b,c^, Krista L. Lanctôt^a,b,c,d^, Nathan Herrmann^b,d,e^, Paul I. Oh^c^, Baiju R. Shah^b,e^, Jeremy Gilbert^e^, Angela Assal^e^, Ilana J. Halperin^e^, Ameer Y. Taha^f,g,h^, Walter Swardfager^a,b,c^*

^a^Department of Pharmacology & Toxicology – University of Toronto, Medical Sciences Building, 1 King's College Circle Room 4207, Toronto, Ontario M5S 1A8, Canada

^b^Sunnybrook Research Institute, 2075 Bayview Avenue, Toronto, Ontario M4N 3M5, Canada

^c^University Health Network Toronto Rehabilitation Institute – Rumsey Centre Cardiac Rehabilitation, 347 Rumsey Rd, East York, Ontario M4G 2V6, Canada

^d^Department of Psychiatry - University of Toronto, 250 College Street 8th floor, Toronto, Ontario M5T 1R8, Canada

^e^Sunnybrook Health Sciences Centre, 2075 Bayview Avenue, Toronto, Ontario M4N 3M5, Canada.

^f^Department of Food Science and Technology, College of Agriculture and Environmental Sciences, University of California, Davis, CA, USA

^g^West Coast Metabolomics Center, Genome Center, University of California - Davis, Davis, CA, USA.

^h^Center for Neuroscience, One Shields Avenue, University of California - Davis, Davis, CA, USA.


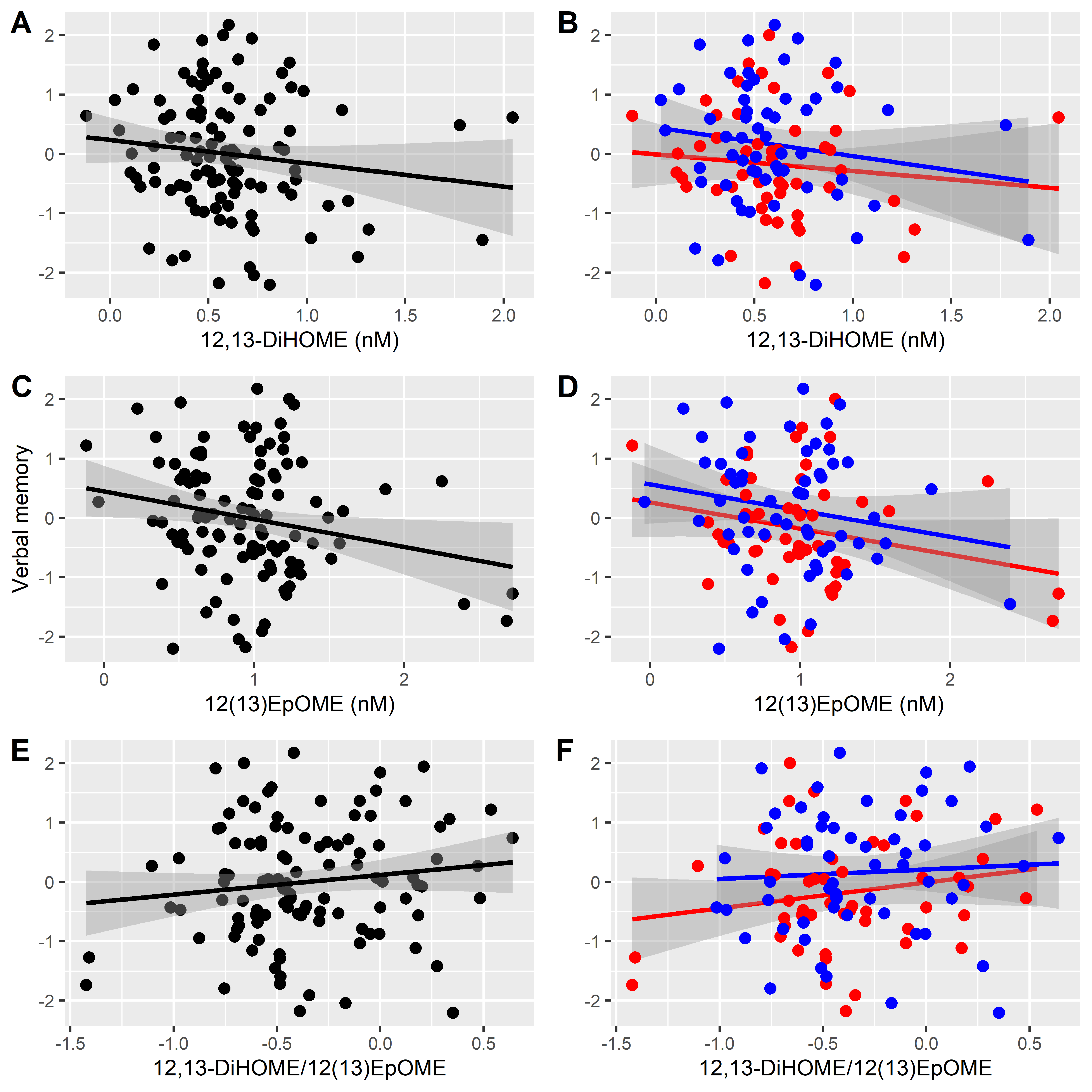


**Supplemental Fig. S1:** Associations between the 12,13-DiHOME species and verbal memory composite score in the **A)** entire group, and **B)** broken down by obese (red) and non-obese (blue) subgroups; associations between the 12(13)EpOME species and verbal memory composite score in the **C)** entire group, and **D)** broken down by obese (red) and non-obese (blue) subgroups; associations between the 12,13-DiHOME/12(13)EpOME ratio and verbal memory composite score in the **E)** entire group, and **F)** broken down by obese (red) and non-obese (blue) subgroups.


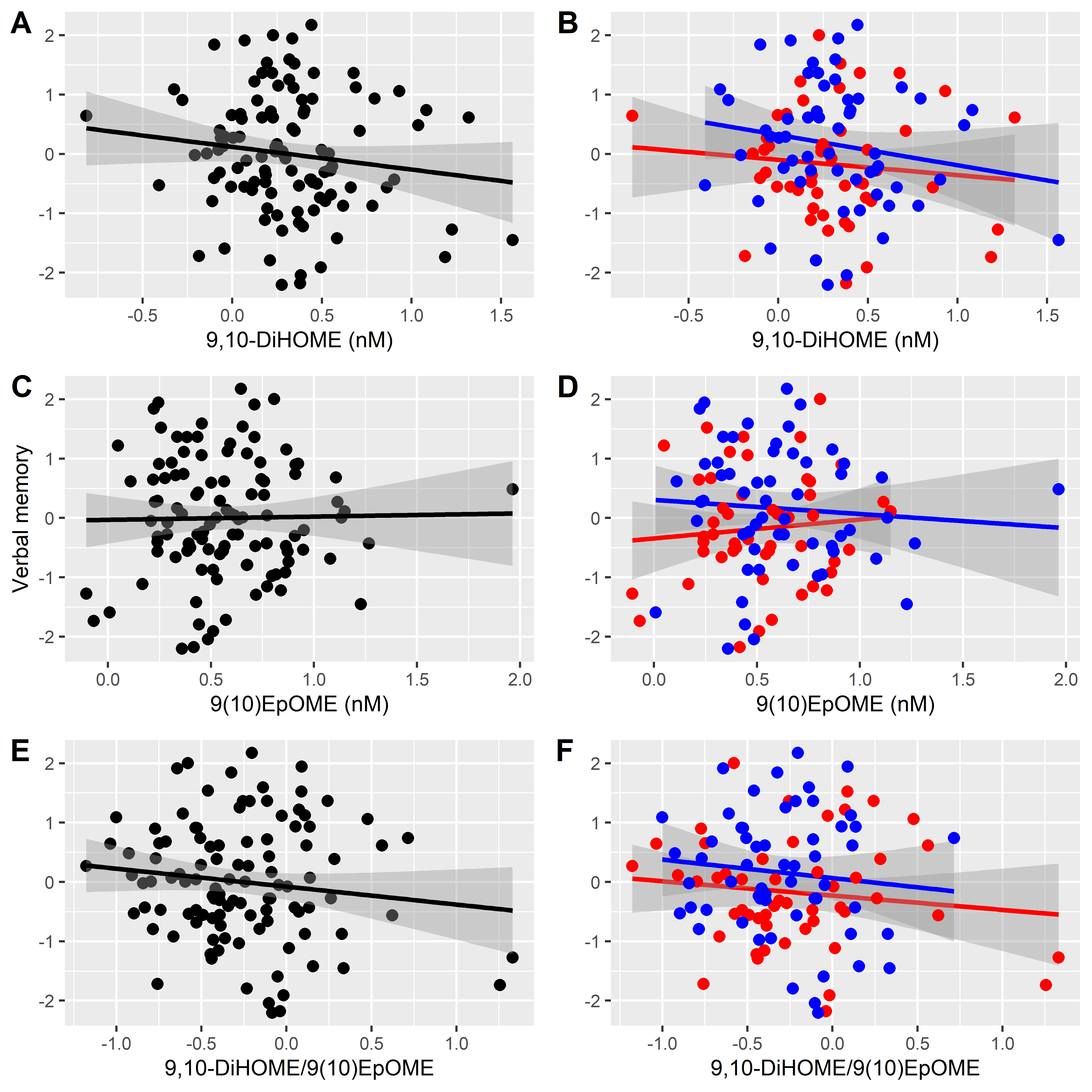


**Supplemental Fig. S2:** Associations between the 9,10-DiHOME species and verbal memory composite score in the **A)** entire group, and **B)** broken down by obese (red) and non-obese (blue) subgroups; associations between the 9(10)EpOME species and verbal memory composite score in the **C)** entire group, and **D)** broken down by obese (red) and non-obese (blue) subgroups; associations between the 9,10-DiHOME/9(10)EpOME ratio and verbal memory composite score in the **E)** entire group, and **F)** broken down by obese (red) and non-obese (blue) subgroups.

**Supplemental Results – Executive Function**

- 1. **Post-hoc analyses controlling for sleep apnea**

**1.1.1 Main analyses**

Because sleep apnea was more common in the obese subgroup, the executive function analyses were repeated after controlling for this covariate.

In a model adjusted for age, sex, BMI, HbA1c, diabetes duration, depression status, hypertension, years of education, and sleep apnea, the 12,13-DiHOME was associated with lower executive function scores (F_1,97_=7.416, p=0.008). The 12(13)-EpOME was also associated with lower executive function scores (F_1,97_=7.162, p=0.009).

No associations were observed for 9,10-DiHOME (F_1,97_=2.968, p=0.088), 9(10)-EpOME (F_1,97_=2.325, p=0.131), the diol/epoxide ratios 12,13-DiHOME/12(13)-EpOME (F_1,97_=0.445, p=0.506) or 9,10-DiHOME/9(10)-EpOME (F_1,97_=0.138, p=0.711).

**1.1.2 Obesity subgroup analyses**

In models adjusted for age, sex, obesity group, HbA1c, diabetes duration, depression status, hypertension, years of education, sleep apnea, and an obesity × oxylipin interaction term, the association between 12(13)-EpOME and lower executive function scores trended more strongly in the obese group (obesity x 12(13)-EpOME interaction F_1,96_=3.433, p=0.067). The 12,13-DiHOME/12(13)-EpOME ratio was associated with higher executive function scores in the obese group (obesity x 12,13 ratio interaction F_1,96_=6.110, p=0.015). The 9(10)-EpOME was associated with lower executive function scores in the obese group (obesity x 9(10)-EpOME interaction F_1,96_=4.274, p=0.041).

No interaction was observed between obesity and 12,13-DiHOME (obesity x 12,13-DiHOME interaction F_1,96_=0.000, p=0.984), 9,10-DiHOME or (obesity x 9,10-DiHOME interaction F_1,96_=0.080, p=0.778) or the 9,10-DiHOME/9(10)-EpOME ratio (obesity x 9,10 ratio interaction F_1,96_=2.032, p=0.157).

- 1. **Post-hoc analyses controlling for antihypertensive use**

**1.2.1 Main analyses**

Because antihypertensive use was more common in the obese subgroup, the executive function analyses were repeated after controlling for this covariate.

In a model adjusted for age, sex, BMI, HbA1c, diabetes duration, depression status, hypertension, years of education, and antihypertensive use, the 12,13-DiHOME was associated with lower executive function scores (F_1,97_=7.436, p=0.008). The 12(13)EpOME was also associated with lower executive function scores (F_1,97_=7.157, p=0.009).

No associations were observed for 9,10-DiHOME (F_1,97_=2.994, p=0.087), 9(10)EpOME (F_1,97_=2.428, p=0.122), the diol/epoxide ratios 12,13-DiHOME/12(13)EpOME (F_1,97_=0.467, p=0.496) or 9,10-DiHOME/9(10)EpOME (F_1,97_=0.132, p=0.717).

**1.2.2 Obesity subgroup analyses**

In models adjusted for age, sex, obesity group, HbA1c, diabetes duration, depression status, hypertension, years of education, anti-hypertensive use, and an obesity × oxylipin interaction term, the association between 12(13)EpOME and lower executive function scores trended more strongly in the obese group (obesity x 12(13)EpOME interaction F_1,96_=3.379 p=0.069). The 12,13-DiHOME/12(13)EpOME ratio was associated with higher executive function scores in the obese group (obesity x 12,13 ratio interaction F_1,96_=5.500, p=0.021). The 9(10)EpOME was associated with lower executive function scores in the obese group (obesity x 9(10)EpOME interaction F_1,96_=4.241, p=0.042).

No interaction was observed between obesity and 12,13-DiHOME (obesity x 12,13-DiHOME interaction F_1,96_=0.002, p=0.969), 9,10-DiHOME or (obesity x 9,10-DiHOME interaction F_1,96_=0.065, p=0.799) or the 9,10-DiHOME/9(10)EpOME ratio (obesity x 9,10 ratio interaction F_1,96_=1.775, p=0.186).

**1.3 Post-hoc analyses excluding cases with missing oxylipin markers**

**1.3.1 Main analyses**

Because the 12,13-DiHOME, 9,10-DiHOME and 9(10)EpOME had some missing cases (see **Table 2** for detectability), the executive function analyses were repeated after excluding these missing samples.

In a model adjusted for age, sex, BMI, HbA1c, diabetes duration, depression status, hypertension, and years of education, the 12,13-DiHOME was associated with poorer executive function composite scores (F_1,95_=6.672, p=0.011). The 9(10)EpOME was also associated with lower executive function composite scores in an adjusted model (F_1,95_=4.415, p=0.038).

No associations were observed for 9,10-DiHOME (F_1,91_=1.894, p=0.172), or the diol/epoxide ratios 12,13-DiHOME/12(13)EpOME (F_1,95_=0.881, p=0.350) or 9,10-DiHOME/9(10)EpOME (F_1,89_=0.911, p=0.342).

**1.3.2 Obesity subgroup analyses**

The 12,13-DiHOME/12(13)EpOME ratio was associated with higher executive function scores in the obese group in the adjusted model (obesity x 12,13 ratio interaction F_1,94_=4.282, p=0.041).

The 9(10)EpOME was associated with lower executive function scores in the obese group (9(10)EpOME x interaction F_1,94_=9.559, p=0.003).

No interactions between obesity were found with 12,13-DiHOME (obesity x 12,13-DiHOME interaction F_1,94_= 0.047, p=0.828), 9,10-DiHOME (obesity x 9,10-DiHOME interaction F_1,90_=0.005, p=0.945) or the 9,10-DiHOME/9(10)EpOME ratio (obesity x 9,10-ratio interaction F_1,88_=3.595, p=0.061).
